# Multiple Liver and Muscle Abscesses and Sepsis with Bacillus pantothenticus in a Leukemia Patient

**DOI:** 10.4274/tjh.2013.0315

**Published:** 2014-09-05

**Authors:** Elif Gülsüm Ümit, Hasan Celalettin Ümit, Figen Kuloğlu, Ahmet Muzaffer Demir

**Affiliations:** 1 Trakya University Faculty of Medicine, Department of Hematology, Edirne, Turkey; 2 Trakya University Faculty of Medicine, Department of Gastroenterology, Edirne, Turkey; 3 Trakya University Faculty of Medicine, Department of Infectious Diseases, Edirne, Turkey

**Keywords:** Leukemia, Infection, Liver abscess

## TO THE EDITOR

Bacilli infections in patients with hematological disorders have been reported, although rare, and subtypes such as Bacillus pantothenticus have been reported in immunocompetent patients with liver abscess [1]. Here we present a case of acute myeloid leukemia and multiple liver and muscle abscesses and sepsis with B. pantothenticus during the neutropenic period, treated successfully with meropenem. Informed consent was obtained.

**Figure 1:**
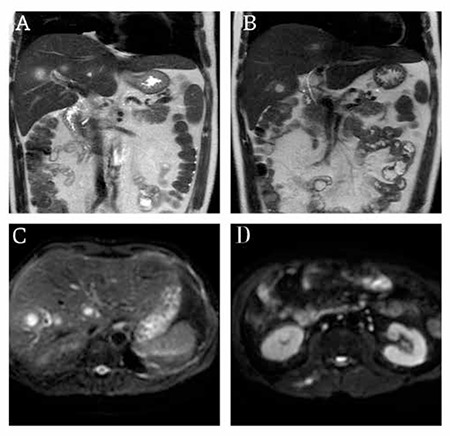
A-B-C: Multiple liver abscesses are seen D: Single focus of abscessin the left paravertebral muscle is seen.

A 49-year-old male patient diagnosed with acute myeloid leukemia was started on remission induction chemotherapy with cytosine arabinoside and idarubicin (7+3). On day 8 of treatment, he had severe neutropenia (40/mm3) and fever (40.0 °C). His physical examination was normal, without organomegaly or tenderness, revealing no hints regarding the source of infection. According to the American Society of Clinical Oncology guidelines for febrile neutropenia, cefepime was started empirically at 2 g 3 times daily after blood and urine samples for culture had been obtained. After 48 h, the patient still had fever (38.3 to 39.4 °C) as tested 6 times a day. The antibiotherapy was switched to meropenem at 1 g 3 times daily following a second set of samples for culture. Fever persisted despite 48 h of meropenem treatment. Serum galactomannan antigen was negative. Thorax computerized tomography revealed no lung infection but multiple masses were seen in the liver (the largest being 3 cm). Magnetic resonance imaging indicated multiple liver abscesses with central necrosis and peripheral contrast enhancement with a single focus of abscess localized in the right paravertebral muscle groups with similar properties (Figure 1).

Percutaneous drainage was performed. A purulent, odorous material was obtained, showing polymorphonucleated neutrophils. The first set of blood cultures of the material remained negative. The second set of cultures was positive for B. pantothenticus susceptible to carbapenems, chloramphenicol, and vancomycin. The first set was incubated for 7 days only, which may be too short for slow-growing bacteria such as B. pantothenticus. On day 28 of meropenem treatment, follow-up magnetic resonance imaging showed no abscesses and only post-inflammatory changes.

Infections with bacilli in patients with hematological disorders have been reported, although subtypes such as B. pantothenticus are extremely rare [2]. During the neutropenic period, abscess formation is not common and it usually occurs after neutrophil recovery.

B. pantothenticus is a rare pathogen probably showing a tendency towards liver and muscle tissues, spreading in a hematogenous fashion; it may cause infection in both immunocompetent and immunocompromised hosts.

Author Contributions: EG Ümit performed the research and wrote the paper, HC Ümit contributed diagnostic tools, F Kuloğlu contributed analytic tools, and AM Demir analyzed and edited the paper.

## CONFLICT OF INTEREST STATEMENT

The authors of this paper have no conflicts of interest, including specific financial interests, relationships, and/ or affiliations relevant to the subject matter or materials included.
